# Experimental challenge of pregnant cattle with the putative abortifacient *Waddlia chondrophila*

**DOI:** 10.1038/srep37150

**Published:** 2016-11-14

**Authors:** Nicholas Wheelhouse, Allen Flockhart, Kevin Aitchison, Morag Livingstone, Jeanie Finlayson, Virginie Flachon, Eric Sellal, Mark P. Dagleish, David Longbottom

**Affiliations:** 1 Moredun Research Institute, Edinburgh, Midlothian, United Kingdom; 2 Biosellal, 317 Avenue Jean Jaurès, 69007 Lyoncedex, France

## Abstract

*Waddlia chondrophila* is a Gram-negative intracellular bacterial organism that is related to classical chlamydial species and has been implicated as a cause of abortion in cattle. Despite an increasing number of observational studies linking *W. chondrophila* infection to cattle abortion, little direct experimental evidence exists. Given this paucity of direct evidence the current study was carried out to investigate whether experimental challenge of pregnant cattle with *W. chondrophila* would result in infection and abortion. Nine pregnant Friesian-Holstein heifers received 2 × 10^8^ inclusion forming units (IFU) *W. chondrophila* intravenously on day 105–110 of pregnancy, while four negative-control animals underwent mock challenge. Only one of the challenged animals showed pathogen-associated lesions, with the organism being detected in the diseased placenta. Importantly, the organism was re-isolated and its identity confirmed by whole genome sequencing, confirming Koch’s third and fourth postulates. However, while infection of the placenta was observed, the experimental challenge in this study did not confirm the abortifacient potential of the organism.

*Waddlia chondrophila* is a *Chlamydia*-related organism originally isolated in 1986 from a bovine foetus in the United States^1^, and was initially described as a *Rickettsia* due to its cross-reactivity with *Cowdria ruminantium* antisera. The organism, which was named WSU 86-1044 was subsequently shown to possess a developmental cycle and replicate within intracellular vacuoles, similar to a group of Gram-negative intracellular bacteria of the order Chlamydiales^2^. Subsequent phylogenetic analysis of the 16 S gene confirmed its position in the order Chlamydiales, and it was named *Waddlia chondrophila*^3^.

A direct causative role in bovine abortion was suggested following its initial isolation from a 3 month old bovine foetus in the United States^1^. *Waddlia chondrophila* was subsequently isolated from a third trimester septic still-born calf in Germany^4^. However, the pathogenic role of the organism in this second case was complicated by the co-isolation of the cattle abortifacient *Neospora caninum*. Several other studies have provided indirect evidence for a role for *W. chondrophila* in reproductive disease in cattle, with serological studies providing limited evidence of an association between *W. chondrophila* antibody titres and pregnancy failure^5^. More recently, studies in North Africa have identified the presence of *W. chondrophila* DNA in vaginal swabs from the dams of aborted bovine fetuses^6^,^7^.

While the original isolations of *W. chondrophila* were from bovine abortions, there has been increasing interest in the role of this organism as a potential zoonotic reproductive pathogen. Recently, *W. chondrophila* has been identified in the placentas of miscarried human pregnancies by both molecular and immunohistochemical methodologies^8^. An association of *W. chondrophila* seropositivity with infertility and adverse pregnancy outcomes has also been suggested in women with tubal factor infertility^9^ and histories of miscarriage^10^, although this is only based on limited serological evidence.

Despite an increasing number of observational studies linking *W. chondrophila* infection to cattle abortion, little direct experimental evidence exists. To date, only one study has investigated the effects of *W. chondrophila* on bovine fetopathy^11^, where direct administration of the pathogen into two bovine fetuses resulted in the death of one of the fetuses within two weeks. But since this study there has been a paucity of direct evidence supporting a role for the organism as an abortifacient. The current study was carried out to investigate whether experimental challenge of pregnant cattle with *W. chondrophila* would result in infection and abortion.

## Results

### Rectal Temperatures

**Figure 1:**
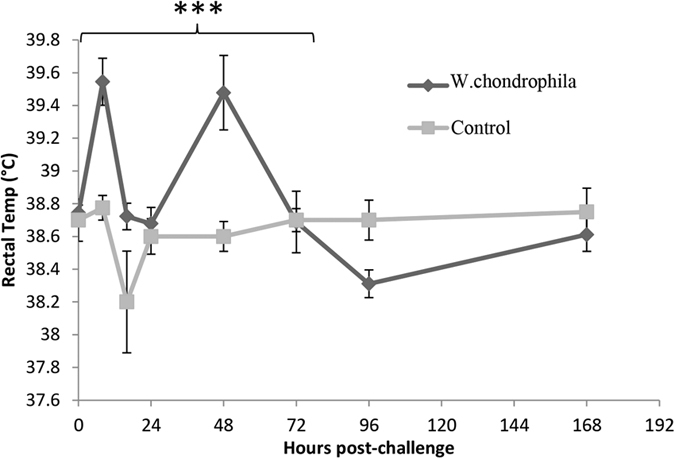
Febrile response of pregnant cattle after challenge with *W. chondrophila*. Animals were challenged with either 2 × 10^8^ IFU *W. chondrophila* or control inoculum at 3 months of pregnancy. Rectal temperatures were obtained from pregnant cattle prior to and post challenge (at 8 hour intervals for first 24 hours then every 24 hours) with either control inoculum (n = 4) or *W. chondrophila* (2 × 10^8^ IFU) (n = 9). Results are presented as mean temperatures ± SEM. ***Significant increase in rectal temperature in *W. chondrophila* infected cattle over the initial 72 h p.c. compared with controls (P = 0.0047).

Temperature data is summarised in Fig. 1. A modest but statistically significant febrile response was observed in the challenged group over the initial 72 hour period post-challenge (p.c.) (0.38 °C higher than the negative control group; p = 0.0047). Conversely, at 96 h p.c. the rectal temperatures of the animals in the challenged group had become significantly lower than those of the negative control group (38.31 °C infected vs. 38.70 °C negative control; p = 0.0362). However, at 168 h p.c. there was no statistically significant difference between both groups (p = 0.3921).

### White blood counts

**Figure 2:**
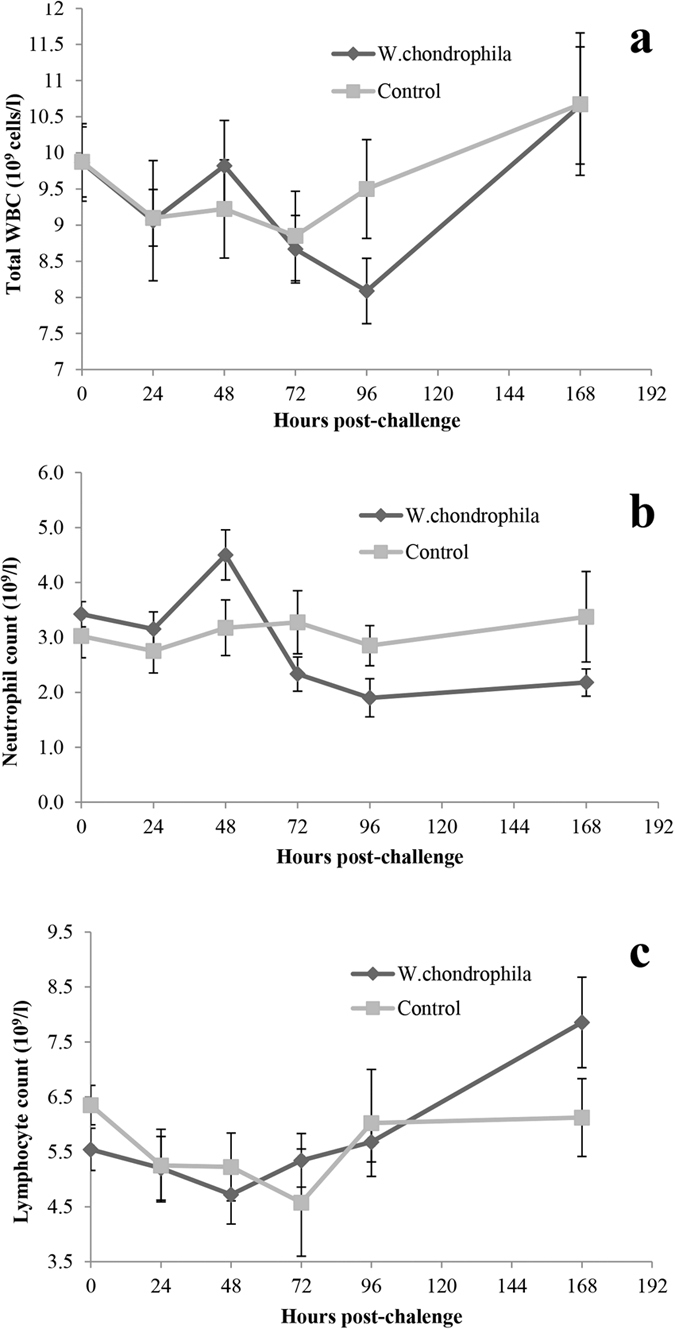
White blood cell counts of pregnant cattle after challenge with either control inoculum or *W. chondrophila*. Blood samples were obtained from pregnant cattle prior to and at specified time points after challenge with either a control (n = 4) or *W. chondrophila* (2 × 10^8^ IFU) (n = 9) and analysed for the presence of (**a**) Total white blood cells (WBC), (**b**) Neutrophils and (**c**) Lymphocytes. Results are presented as Mean counts ± SEM. No significant differences were observed in cell counts between groups.

It has been reported previously that *W. chondrophila* challenge of goats and calves resulted in a transient leukopenia^11^, therefore white blood cell (WBC) counts were performed to determine the effects of *W. chondrophila* challenge in adult pregnant cattle (Fig. 2). Total WBC counts at the time of challenge were initially the same in both treatment groups (9.9 × 10^9^ ± 0.5 × 10^9^ cells/L negative control vs 9.9 × 10^9^ ± 0.5 × 10^9^ cells/L challenge group) (Fig. 2a). There were no significant differences in WBC counts between groups over the initial 72 h period (p = 0.936). While there was an apparent reduction in total WBC numbers by 96 h in the challenge group this was not significant (9.5 × 10^9^ ± 0.7 × 10^9^ cells/L negative control vs 8.1 × 10^9^ ± 0.5 × 10^9^ cells/L challenge group; p = 0.0892). Numbers of WBC were again identical at 168 h p.c. (10.7 × 10^9^ ± 1.2 × 10^9^ cells/L negative control vs 10.7 × 10^9^ ± 0.7 × 10^9^ cells/L challenge group; p = 0.6993).

Consistent with total WBC counts there were no significant differences in neutrophil numbers within either the initial 0–72 h p.c. (p = 0.7830), or at 96 h p.c. (2.9 × 10^9^ ± 0.7 × 10^9^ cells/L negative control vs 1.9 × 10^9^ ± 1.0 × 10^9^ cells/L challenge group; p = 0.141) (Fig. 2b). At 168 h p.c. there was an apparent decrease in neutrophil count in the challenged group however this was not significant (3.4 × 10^9^ ± 1.6 × 10^9^ cells/L negative control vs 2.2 × 10^9^ ± 0.7 × 10^9^ cells/L challenge group; p = 0.0752). Lymphocyte counts were also similar across both groups for the 168 h period under study (Fig. 2c). There was no significant difference between groups over the initial 0–72 h period (p = 0.7110), 96 h p.c. and (6.0 × 10^9^ ± 1.9 × 10^9^ cells/L negative control vs 5.7 × 10^9^ ± 1.1 × 10^9^ cells/L challenge group; p = 0.6969), or 168 h p.c. (6.1 × 10^9^ ± 1.4 × 10^9^ cells/L negative control vs 7.9 × 10^9^ ± 2.5 × 10^9^ cells/L challenge group; p = 0.1416).

### Serology

**Figure 3:**
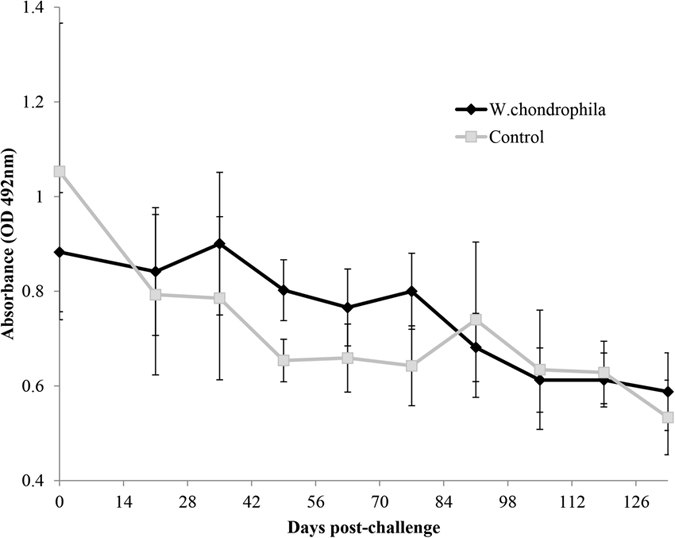
Serological IgG responses of pregnant cattle after challenge with either control inoculum or *W. chondrophila*. Serum samples were obtained from pregnant cattle prior to and at specified time points after challenge with either a control (n = 4) or *W. chondrophila* (2 × 10^8^ IFU) (n = 9) and analysed for the presence of anti-*W. chondrophila* IgG by ELISA. Results are presented as Mean absorbance (at 492 nm) ± SEM. No significant differences were observed in cell counts between groups.

There were no apparent differences in IgG response to the challenge with *W. chondrophila* (Fig. 3). Although the mean IgG responses in the challenged group appeared higher than the control group between 21 and 91d p.c., there was no significant difference overall between groups (p = 0.7692).

### Pathology and Immunohistochemistry

**Figure 4:**
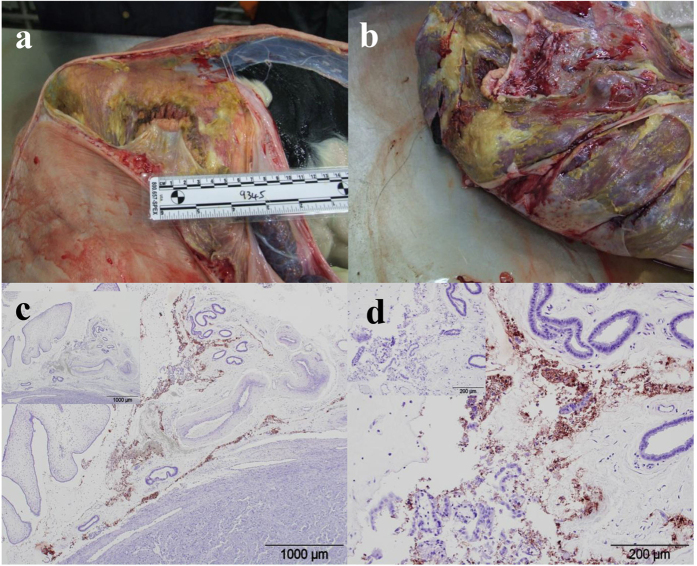
Gross pathology and Immunohistochemical analysis of *W. chondrophila* infected bovine placenta. (**a**) and (**b**) Photographs taken at necropsy demonstrating the presence of a yellow semi-solid exudate covering the surface of the placental chorioallantois of fetus 9345. (**c**) Immunohistochemical detection of *W. chondrophila* antigen on the surface of the chorioallantois, using specific antiserum. (x4 objective), (**d**) x20 objective. Negative control sections in which the primary antibody was replaced with an isotype control antibody are shown (inset).

Gross examination of all nine challenged pregnant heifers identified a single dam (No. 9345, which was euthanased two weeks prior to expected date of parturition) as having a large number of irregularly shaped, coalescing foci comprised of large amounts of yellow, semi-solid exudate on the surface of the chorioallantois in the inter-cotyledonary zones, which occasionally extended onto the outer surface of the placentome (Fig. 4a,b). No significant gross lesions were found in the remaining eight challenged animals or in any of the negative control group. Histological examination of all the tissues from all the dams and fetuses/calves showed a large number of macrophages and fewer neutrophils in the sub-epithelial tissue of the intercotyledonary placental membrane (chorioallantois) of the animal with the gross lesions (No. 9345). Additionally, foci of intense neutrophil infiltration were present within the intercotyledonary membrane directly adjacent to the placentome and small numbers of medium to large sized foci of coagulative necrosis of tissue of fetal origin and large areas of haemorrhage were present between the overlying chorioallantois and the arcade region of the placentome. No significant lesions were present in any of the other tissues examined from dam and fetus no. 9345. Of the remaining eight challenged animals, two fetuses (Nos 9126 and 3307) had mild, focal dysmorphogenesis in the brain; one affecting the hippocampus and cerebellum, the other the cerebral cortex but these lesions were only apparent on histological examination and there were no other significant lesions. One calf (No. 9480) had small to medium numbers of lymphocytes and plasma cells within the meninges overlying the cerebrum and ventral medulla but no other lesions. All *W. chondrophila* challenged dams which were allowed to progress to parturition had some degree of mixed, predominantly suppurative inflammation within their caruncles not untypical of post-parturient cattle. Of the two negative control dams allowed to progress to parturition, one had mild inflammation of the caruncles characterised by infiltration of small numbers of neutrophils and lymphocytes and the other minimal levels of similar inflammation.

Immunohistochemistry on placentomes/caruncles/cotyledons of all animals showed the presence of *Waddlia* antigens in the animal with gross lesions (No. 9345) only. Large amounts of *Waddlia* antigen were present on the surface of the chorioallantois, corresponding with the gross lesions, with little present beneath the epithelial surface (Fig. 4c,d). No *W. chondrophila* antigen was found by IHC in any of the remaining fetal tissues examined (brain, lung, heart, liver, spleen) for this animal.

### Real-time PCR

*Waddlia chondrophila* DNA was detected using a diagnostic real-time PCR in pooled fetal tissues from the heifer with the gross lesions in the foetal membranes, with no detectable DNA identified in any of the maternal or fetal tissues from the remaining animals (4 negative controls and 8 *W. chondrophila* challenged animals). PCR amplification of *W. chondrophila* DNA from each of the individual tissues from the positive pooled sample was observed in all fetal tissues tested, except the liver. As an internal positive control, detection of host glyceraldehyde-3-phosphate dehydrogenase gene (GAPDH) was performed on each sample analysed. The PCR reactions were validated by the successful amplification within the expected Cq range of *W. chondrophila* and GAPDH positive control templates provided with the kit. The Cq values from the fetal samples are summarised in Table 1. There was no detectable *W. chondrophila* DNA in any of the maternal tissues tested (liver, spleen, lung). The highest levels of *W. chondrophila* DNA were observed in the placentomes (1–6; Cq 16.8, 7–12; Cq 20.2), and the lung also exhibited notable levels (Cq 26.7). *Waddlia chondrophila* DNA was also present in the amniotic fluid (Cq 31.7) and fetal stomach contents (Cq 35), albeit at relatively low levels.

### Re-isolation and DNA sequencing of *in-vivo* passaged *W. chondrophila*

**Figure 5:**
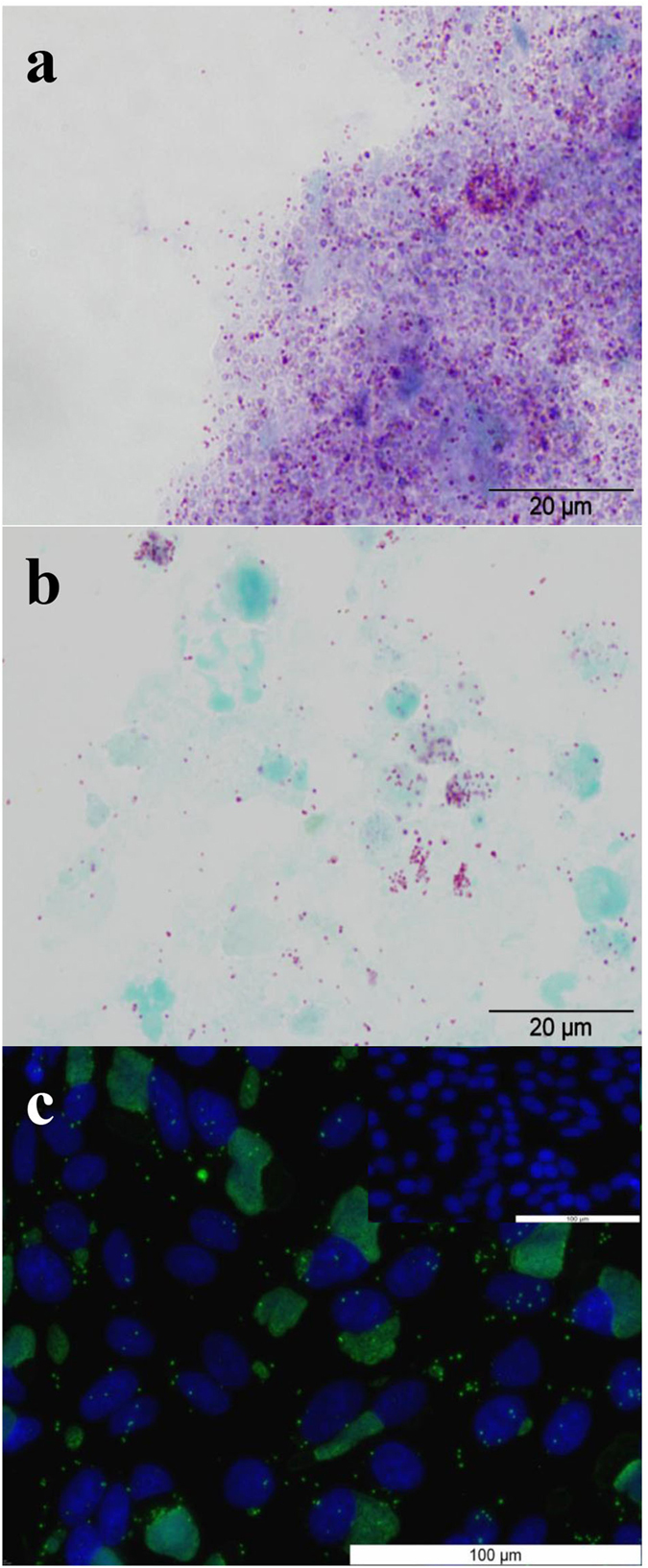
Detection and re-isolation of *W. chondrophila* from experimentally infected bovine placenta. (**a**) mZN detection of large numbers of gram negative cocci (pink) from the placental exudate of fetus 9345. (**b**) mZN of an impression smear from a placental cotyledon of fetus 9345. (**c**) Fluorescent detection of *W. chondrophila* in HEp2 cells infected with organisms re-isolated from the infected bovine placenta (fetus 9345), using specific anti-*W. chondrophila* antisera.

Modified-Ziehl Nielsen (mZN) staining and microscopy of the placental exudate (Fig. 5a) and tissue samples (Fig. 5b) from animal 9345 revealed a high abundance of Gram-negative cocci, similar in shape to, but larger than classical, *Chlamydiaceae* sp. Further culture of this placental exudate material on HEp2 cells *in vitro* revealed the presence of *Chlamydia*-like organisms with the capacity to form cellular inclusions and induce cell lysis; a growth phenotype identical to that of the *W. chondrophila* strain ATCC VR-1470 that was used to challenge the animals. Visualisation of these inclusions at 24 h p.c. with the anti-*W. chondrophila* antisera identified the organisms as *W. chondrophila* (Fig. 5c). Genotypic verification by whole genome sequencing unequivocally established these isolated organisms as being *W. chondrophila* strain ATCC VR-1470 (Whole Genome Shotgun project has been deposited at DDBJ/ENA/GenBank under the accession LVEB00000000. The version described in this paper is version LVEB01000000).

## Discussion

While *W. chondrophila* is regarded as an emerging cattle abortifacient, there has been little direct experimental evidence to demonstrate a causal role in bovine abortion. The only previous study investigating bovine fetopathy following *W. chondrophila* challenge involved direct administration of the organism into two near-term bovine fetuses *in utero* resulting in the death of one of the animals^11^. In this study we have demonstrated the presence of *W. chondrophila* DNA in the placenta, amniotic fluids and fetus in a near-term heifer, and critically, viable organisms in the placenta, 6 months after intravenous infection. This study therefore demonstrates dissemination and the possibility of persistence of infection in the dam, similar to other well characterised chlamydial infections such as *Chlamydia abortus*.

The experimentally induced infection in animal 9345 resulted in the presence of a waxy, yellow exudate across a proportion of the surface of the placenta. This is similar to experimental infection of cattle with *C. abortus* in which an accumulation of yellow-brown exudate between the uterine and chorionic surfaces was observed^12^, and *in vitro* evidence demonstrating a similar pro-inflammatory response to infection with either organism in ovine trophoblasts *in vitro*^13^,^14^. However in the current study, the presence of the *W. chondrophila* and the associated gross lesions were restricted to the surface of the placenta and little pathology or inflammation was observed within the deeper structures of this organ. This contrasts with *C. abortus* infection which results in necrotising placentitis and vasculitis present throughout the full thickness of the inter-cotyledonary areas and deep within the cotyledon tissue also. It has been suggested from the observed differences in the intracellular inclusion formation, substrate sequestration and the absence of anti-apoptotic activity of *W. chondrophila* compared to *C. trachomatis* that the organism may be perhaps more akin to a facultative rather than obligate pathogen in mammalian cells, in contrast with other classical chlamydial species^15^. These differences have been suggested to reflect a reduced level of pathogenicity, and explain the sporadic identification of *W. chondrophila* associated with clinical disease. Of the 9 animals that received the same infective dose of *W. chondrophila* in the current study, the organism could only be identified in 1 animal consistent with this hypothesis.

While the results of the current study demonstrate placental and fetal infection, there was no evidence of abortion. These results would suggest that *W. chondrophila* is likely a facultative pathogen in cattle, which is consistent with its sporadic identification in cases of cattle abortion. However, as with any infection model, the background of the animal and the timing, dose and route of infection can be critical to the outcome. The heifers used in this study were of a high health status and were obtained prior to their entry into adult herds. Although there was no evidence of *W. chondrophila* in any of the animals except after challenge, due to the possible endemic nature of chlamydial organisms within cattle in certain regions^16^ we cannot rule out the possibility that prior exposure to similar organisms may have affected the response to any subsequent infection. The challenge dose used in the current study was similar to that used previously to infect near term bovine foetuses with *W. chondrophila*^11^ and experimental investigations of chlamydial abortion in cattle^17^,^18^,^19^. However, determining the optimal experimental dose for chlamydial challenge infections can be complex and in a recent study we observed that sheep abortion rates were greater with lower (<10^5^ IFU) rather than higher (10^7^ IFU) challenge doses of *C. abortus*^20^. The timing of the infection could also be important in determining experimental outcome. In the current study the animals were challenged at 105–110 days of gestation, a time when the immune system of the fetus is starting to become competent and the brain is in the latter stages of its development. While this may have resulted in the histologically observed small amount of brain dysmorphogenesis in two of the *W. chondrophila* challenged animals these calves had no gross brain lesions and would probably have been clinically normal. However, at present it is difficult to ascertain if there is a critical point in gestation during which the fetus would be more susceptible to infection with *W. chondrophila*. Experimental infection with *C. abortus* in sheep consistently induces late-term abortion in a susceptible proportion of animals, whether the organism is administered during or prior to pregnancy^19^. However, experimental chlamydial infections in cattle suggest a higher degree of variability in the timing of abortion relative to infection, irrespective of the gestational period of infection^17^,^18^, with re-isolation of the organism from an aborted placenta demonstrated as early as day 130 of gestation^16^. The two cases in which *W. chondrophila* has been isolated from field samples were from a 3 month gestation fetus^1^ and late gestation (day 228) septic stillborn calf, when the organism was isolated in conjunction with *Neospora caninum*^4^, and the only previous experimental challenge study of *W. chondrophila* investigating its role in bovine fetopathy was conducted in a late gestation fetus^11^.

This study has demonstrated the lesion-associated presence of the organism in the placenta of an experimentally challenged dam. Importantly, the organism was re-isolated and its identity confirmed by full length genome sequencing, confirming Koch’s third and fourth postulates. However, despite lesion-associated infection of the placenta, the experimental challenge in this study did not confirm the abortifacient potential of the organism as this particular animal was euthanized prior to parturition. Nor was the organism identified in any of the other remaining 8 challenged animals, suggesting that at least in terms of the current experimental design, the pathogenicity of the organism with regard to abortion was limited. Given the current findings, which demonstrate placental infection but also the paucity of information on *W. chondrophila* infection in cattle, additional experimental studies are required to further examine the abortifacient potential of the organism and which, if any, factors influence this.

## Materials and Methods

### Ethics Statement

This study was carried out in strict accordance with the UK Animals (Scientific Procedures) Act 1986 and in compliance with all UK Home Office Inspectorate regulations. The experimental protocol was approved by the Moredun Experiments and Ethical Review Committee (Permit number: E35/14). All animals were monitored throughout the 6 month study for any clinical signs of disease at least three times daily and all findings recorded. Any animal found to be suffering or requiring treatment was given appropriate veterinary care in accordance with standard veterinary practice.

### Preparation of inoculum

*Waddlia chondrophila* strain ATCC VR-1470 was grown at 37 °C in HEp2 (ECACC, Salisbury, UK) cells cultured in GIBCO^®^ GlutaMAX Iscove’s Modified Dulbecco’s Medium (IMDM) (Thermo Fisher Scientific, Renfrew, UK) as previously described^14^. Media was supplemented with 2% heat inactivated fetal calf serum (PAA Laboratories Ltd, Yeovil, Somerset, UK) and 1 μg/ml cycloheximide (Sigma-Aldrich, Poole, UK). The inoculum was titrated on 8-well chamber slides (BD Falcon, Becton Dickinson, Bedford, UK) and visualised according to a previously published protocol^14^ using polyclonal antisera raised against *W. chondrophila* elementary bodies (a gift from Professor Gilbert Greub, University of Lausanne).

### Animals

Thirteen healthy pregnant Friesian Holstein Heifers (bovine viral diarrhoea virus and bovine herpesvirus-1 free) at 3 months gestation were purchased from farms in Denmark. Following transportation the animals were given a 2 week acclimatisation period prior to challenge. Nine of the animals received 2 × 10^8^ inclusion forming units (IFU) *W. chondrophila* in a total volume of 5 ml sucrose–phosphate–glutamate (SPG) buffer^21^ via the jugular vein. Four negative-control animals underwent mock challenge substituting the same volume of vehicle for the inoculum. Animals were monitored closely over the initial 24 hour period post-challenge (p.c.) with rectal temperatures and blood obtained at 4 hourly intervals for routine haematology (analysis performed commercially by SAC Consulting, Penicuik, Midlothian, UK). Pregnancy was confirmed and monitored by ultrasound scanning *per rectum* which was performed after initial acclimatisation and at weekly intervals over the course of the study.

Two negative control and 4 *W. chondrophila* challenged animals were euthanized 2 weeks prior to the date of parturition, calculated from the known date of artificial insemination, to allow the collection of aseptic tissues for molecular analysis and recovery of the organism. The remaining animals (2 negative control and 5 challenged animals) were allowed to proceed to, and undergo, parturition. The placenta was obtained at the earliest opportunity after natural expulsion and the mother and calf were euthanized within 48 hours. Adult cattle were euthanized by exsanguination after stunning using a captive bolt. Calves were euthanized by intravenous administration of pentobarbitone sodium B.P. (approx. 200 mg/kg; Rhone Merieux, England).

Weight and crown-rump length were obtained for each fetus/calf prior to necropsy. The intact thyroid was dissected free of surrounding material and weighed. At necropsy all tissue samples that were to be analysed by DNA analysis were taken with individual clean, sterile, single use scalpels and forceps and placed in sterile polystyrene universal tubes for storage at −80 °C. Tissue samples for histopathology (Dam: left tonsil, lung, heart, liver, spleen, distal mesenteric lymph node, kidney, ovaries, uterine lymph nodes, placentome/caruncles [10 samples], mammary gland and pre-femoral lymph nodes. Fetus/calf: whole brain, left tonsil, left retropharyngeal lymph node, thyroid, thymus, lung, heart, diaphragm, liver, spleen, distal mesenteric lymph node, kidney, semitendinosus skeletal muscle, left pre-femoral lymph node and cotyledons (10 samples) were trimmed and placed in 10% neutral buffered formalin.

The uterus (including fetus) and cervix were removed intact from each animal that was euthanized prior to parturition. Sterile amniotic fluid was obtained prior to excision of the fetal membranes and removal of the fetus. Vaginal tissue for histopathology and dry swab samples for PCR were taken from the external orifice of the uterus prior to careful excision of the uterine wall. A dry swab sample was taken from the internal orifice of the uterus and cervical tissue obtained for histopathology. A total of 10 placentomes were obtained from each placenta. Each placentome was split into 2 equal-sized samples, one sample trimmed and fixed for histopathology and the other half pooled for DNA analysis. Similar samples were also taken for the preparation of smears for staining using the mZN method^22^. Smears were visualised under high-power microscopy for the presence of *W. chondrophila* elementary bodies (EBs) and other contaminating bacteria. Fetal stomach contents were removed aseptically using a sterile needle and syringe prior to collection of remaining tissue samples.

For animals that underwent parturition, cotyledons rather than intact placentomes were obtained from the expelled placentas and caruncles from the uterus for both histopathology and DNA extraction. Amniotic fluid and fetal stomach contents could not be guaranteed free from environmental contamination in these animals and were, therefore, not taken for molecular studies.

### Serology

Serum samples for serology were obtained prior to challenge (Day 0), at 3 weeks post challenge (Day 21), and then at fortnightly intervals throughout the duration of the study (Day 35, 49, 63, 77, 91, 105 & 119); with a final sample obtained on the day of euthanasia (Day 133). A semi-quantitative ELISA was developed to determine differences in the relative titre of IgG antibodies against *W. chondrophila* between animals pre and post-challenge. Due to the paucity of defined positive control serum, backgrounds were determined and optimised using newborn and normal adult cattle sera and the lack of cross-reactivity was determined using defined sera obtained from *C. abortus* and *Chlamydia pecorum* challenged cattle. ELISA wells (96 well Maxisorb ELISA plate; Nunc) were coated with formalin inactivated (0.5% v/v final concentration) *W. chondrophila* EBs diluted in carbonate bicarbonate buffer (pH 9.6; Sigma C3041) to an OD600 nm of 0.2; and incubated overnight at 4 °C. Plates were washed four times in wash buffer (1 x Tris-Buffered Saline (TBS) (Sigma T5912) + 0.05% v/v Tween-20) and blocked with 3% w/v soya diluent (SMA Wysoy)/TBS for 1 hour at 37 °C before washing a further 4 times with wash buffer. Serum samples diluted at 1:1000 in sample diluent (1% w/v soya in TBS + 0.05% Tween-20) were analysed in duplicate and incubated for 1 hour at 37 °C before washing 4 times with wash buffer. Rabbit-anti-bovine IgG:HRP conjugated antibody (Sigma; A5295) was diluted 1:1000 in sample diluent and incubated for 1 hour at 37 °C before washing a further 4 times. The reaction was detected by incubating with OPD peroxidise substrate (Sigma-fast tablets, P9187) for 30 minutes, the reaction was stopped with 3 M H_2_SO_4_ and the plates read at OD 492 nm.

### DNA extraction from tissues

DNA was extracted from maternal and fetal or calf tissue pools comprising placenta, liver, spleen, amniotic fluid and fetal stomach contents (from preterm foetuses only) using the Qiagen DNeasy^®^ Blood and Tissue kit (Qiagen Ltd., Manchester, UK) as per the manufacturer’s instructions. DNA was eluted in a 200 μl final volume of AE buffer. Where *W. chondrophila* DNA was detected in any tissue pool, individual tissues and fluids were analysed. Negative extraction controls were carried out on each extraction run.

### Real-time PCR

The presence of *W. chondrophila* DNA in tissue samples and an internal positive control (GAPDH) were detected using a specific real-time PCR (prototype Bio-T^®^ *Waddlia chondrophila*, Biosellal, Lyon, France). Briefly, 5 μl of extracted DNA was added to 15 μl premixed Mastermix containing primers and probe. Positive controls for *W. chondrophila* and internal positive control were supplied with the kit. Non-template controls consisted of molecular grade water substituted for the DNA sample. Samples and controls were analysed in duplicate and the assay was run on an Applied Biosystems ABI 7500 real-time PCR system (Thermo Fisher Scientific, Renfrew, UK). The specificity of the assay was demonstrated against a panel of chlamydial species (kindly supplied by Professor Gilbert Greub, University of Lausanne, Switzerland) and clinically derived non-chlamydial bacterial species (S1 Table).

### Histology and immunohistochemical analysis

All neutral buffered formalin fixed samples were prepared for histopathological examination by standard techniques (dehydrated through graded alcohols, embedded in paraffin wax, sectioned [5 μm], mounted on glass slides and stained with haematoxylin and eosin [HE]). Duplicate sections from the placentome/caruncle/cotyledon samples were also subjected to specific immunohistochemistry (IHC) for *W. chondrophila* antigen. After dewaxing in xylene and rehydration through graded alcohols, sections were incubated with 3% H_2_O_2_ in methanol (v/v) for 20 minutes at room temperature (RT) to quench endogenous tissue peroxidase activity and washed in phosphate-buffered saline (PBS) prior to antigen unmasking in a solution of 0.01% Pronase (Roche Diagnostics Ltd., Burgess Hill, West Sussex) in PBS for 10 minutes at RT. After three washes in PBS containing 0.05% Tween_20_ (PBS-T), non-specific antibody binding was blocked using Protein Block Serum Free (Dako, UK Ltd, Ely, Cambridgeshire UK) for 30 minutes at RT, prior to incubation overnight at 4 °C with a 1:2000 dilution of rabbit polyclonal antisera raised against *W. chondrophila* EBs, in antibody diluent with background reducing agents (Dako). Following three further washes in PBS-T, bound antibody was visualised using the Dako EnVision^™^ System-HRP (K4011, Dako) and ImmPACT^™^ NovaRED (Vector Labs, Peterborough, UK) chromogen. Sections were washed in water, counterstained with haematoxylin, dehydrated through graded alcohols, cleared and mounted. The positive control sample consisted of formalin fixed, routinely processed and wax embedded *W. chondrophila* infected HEp2 cells. Negative control reactions were performed for each sample in which normal rabbit serum was substituted for the primary antiserum. All tissue sections were examined by light microscopy for the presence and distribution of lesions and *W. chondrophila* antigens.

### Re-isolation and DNA sequencing of *in-vivo* passaged *W. chondrophila*

A surface swab of the foetal membranes was taken and the head of the swab snapped off into a bijou containing 1 mL of sterile PBS. Following rigourous vortexing, 0.5 ml of the material was inoculated onto sub-confluent *Acanthamoeba castellanii* cells (gifted by Dr Sutherland Maciver, The University of Edinburgh) at 25 °C, in amoeba isolation media (14.3 g/L peptone (Oxoid), 7.15 g/L yeast extract (BD), 15.4 g/L glucose, 0.51 g/L Na_2_HPO_4_, 0.486 g/L KH_2_ PO_4_, 1 μg/ml cycloheximide 50 μg/ml gentamicin, 200 μg/ml streptomycin and 25 U/ml nystatin; all at final concentration); and HEp2 cells at 37 °C, in IMDM (supplemented with 2% heat inactivated fetal calf serum, 1 μg/ml cycloheximide, 50 μg/ml gentamicin, 200 μg/ml streptomycin and 25 U/ml nystatin). Following incubation for 7 days, characteristic *Chlamydia*-like inclusions could be observed in both the HEp2 and *A. castellanii* cells, and the organisms were isolated and stored as described^14^.

For whole genome sequencing, 3 x T225 flasks of infected *A. castellanii* were harvested using sterile glass beads and centrifuged at 153 × *g* (JLA-16.250 rotor) for 10 min at 4 °C to remove gross cellular debris. Cell pellets were washed in ice-cold PBS and re-centrifuged at 153 × *g*, the supernatants removed and centrifuged at 22,100 × *g* (JLA-16.250 rotor) for 30 min at 4 °C. Each pellet was re-suspended in 18 ml 20 mM Tris-HCl, pH 7.5/150 mM KCl/1% sarkosyl and homogenised lightly using a ground glass homogeniser. Homogenised cells were layered onto 3 ml sucrose cushions (15% sucrose in 20 mM Tris-HCl, pH 7.5/150 mM KCl/1% sarkosyl) and centrifuged at 70,000 × *g* (Beckman Coulter SW40 rotor) for 45 min at 4 °C. DNA was prepared using the DNeasy^®^ Blood and Tissue kit (Qiagen) as per the manufacturer’s instructions. The DNA was eluted into a final volume of 0.5 ml molecular grade water (Sigma) and quantified (total DNA concentration of 123.7 ng/μl) using a NanoDrop spectrophotometer (ND-1000). A total volume of 20 μl isolated DNA was then submitted for genome sequencing by MicrobesNG (University of Birmingham, UK).

### Statistics

A generalised additive mixed model (GAMM) was fitted by restricted maximum likelihood (REML), including group (infected, control) as fixed effect and animal ID as random effect. To model rectal temperature and blood cell counts the statistical analysis was performed independently upon the data over 3 distinct time periods, from 0 to 72 hours post-challenge (p.c.), and at 96 h and 168 h p.c. These later two time-points were considered distant enough to treat them as independent. Trends over time were estimated by individual smoothing splines per group and considering homogeneous within-group error variances. A non-parametric Mann-Whitney test was used to analyse individual time points at 96 h and 168 h p.c.

## Additional Information

**Accession codes:** Whole Genome Shotgun project has been deposited at DDBJ/ENA/GenBank under the accession LVEB00000000. The version described in this paper is version LVEB01000000. 

**How to cite this article**: Wheelhouse, N. *et al*. Experimental challenge of pregnant cattle with the putative abortifacient *Waddlia chondrophila*. *Sci. Rep*. **6**, 37150; doi: 10.1038/srep37150 (2016).

**Publisher’s note**: Springer Nature remains neutral with regard to jurisdictional claims in published maps and institutional affiliations.

## Supplementary Material

Supplementary Information

## Figures and Tables

**Table 1:** Individual tissue Cq values for fetus 9345.

Sample	Quantitation cycle (Cq)	
	*W. chondrophila*	GAPDH
Placentome 1–5	16.8	18.6
Placentomes 6–10	20.2	19.7
Liver	Neg	16.8
Lung	26.7	23.3
Spleen	33.9	27.4
Aminotic fluid	31.7	22.7
Fetal Stomach Contents	35.0	23.0
