# Supplementary material for: Experimental challenge of pregnant cattle with the putative abortifacient Waddlia chondrophila
Source: Sci Rep. 2016 Nov 14;6:37150. doi: 10.1038/srep37150 (PMC5107887; doi:10.1038/srep37150)
Supplement: Supplementary Information [file srep37150-s1.pdf]

Experimental challenge of pregnant cattle with the putative abortifacient *Waddlia chondrophila*

Author List

Nicholas Wheelhouse[1# ¶ *], Allen Flockhart[1# ¶], Kevin Aitchison[1], Morag Livingstone[1], Jeanie Finlayson[1], Virginie Flachon[2], Eric Sellal[2], Mark P. Dagleish[1] & David Longbottom[1]

Supplementary Table 1. List of bacterial species and ruminant pathogens used to determine the specificity of the BioSellal *Waddlia chondrophila* qPCR

Species	Amplification
**Chlamydial species**	
Parachlamydia acanthamoebae (Hall's coccus)	-
Parachlamydia acanthamoebae (BN9)	-
Waddlia chondrophila	+
Protochlamydia naegleriophila (Knic)	-
Protochlamydia amoebophila (UWE25)	-
Estrella lausannensis	-
Simkania negevensis	-
Neochlamydia hartmanellae	-
Criblamydia sequanensis	-
Chlamydia abortus	-
Chlamydia pecorum	-
Chlamydia psittaci	-
**Non-chlamydial bacterial species**	
Serratia marcescens	-
Klebsiella oxytoca	-
Pseudomonas aeruginosa	-
Escherichia coli	-
Borrelia burgdoferi	-
Salmonella Dublin	-
Salmonella Typhimurium	-
Coxiella burnetii	-
Pasteurella multocida	-
Listeria monocytogenes	-
Arcanobacter pyogenes	-
Staphylococcus aureas	-
Staphylococcus haemolyticus	-
Staphylococcus chromogenes	-
Staphylococcus intermidius	-

Staphylococcus epidermis	-
Streptococcus pyogenes	-
Streptococcus parauberis	-
Streptococcus bovis	-
Streptococcus dysgalactiae	-
Streptococcus canis	-
Enterococcus faecium	-
Enterobacter aerogenes	-
Enterobacter absuriae	-
Enterobacter lignotyticus	-
Raoultella ornithinolytica	-
Corynebacterium bovis	-
Aerococcus viridans	-
Mycoplasma bovis	-
Brucella melitensis	-
Mycobacterium avium paratuberculosis	-
Leptospira interrogans	-
**Non–bacterial ruminant pathogens**	
Toxoplasma gondii	-
Neospora caninum	-
Bovine Viral Disease virus-1	-
Border Disease-4	-
Bovine Herpes Virus-1	-
Bovine Herpes Virus-4	-
	-
**Host-species for chlamydial growth**	
Acanthamoebae castellanii (ATCC 30010)	-
